# Vagal cross-sectional area correlates with parasympathetic dysfunction in Parkinson's disease

**DOI:** 10.1093/braincomms/fcad006

**Published:** 2023-01-18

**Authors:** Sophie Huckemann, Katharina Mueller, Paulina Averdunk, Eva Kühn, Lovis Hilker, Saskia Kools, Leonard Scholz, Yesim Bulut, Jil Brünger, Sean Fiegert, Thomas Grüter, Anna Lena Fisse, Jeremias Motte, Min-Suk Yoon, Ralf Gold, Christiane Schneider-Gold, Lars Tönges, Kalliopi Pitarokoili

**Affiliations:** Department of Neurology, St. Josef Hospital, Ruhr-University Bochum, Bochum, Germany; Department of Neurology, St. Josef Hospital, Ruhr-University Bochum, Bochum, Germany; Department of Neurology, St. Josef Hospital, Ruhr-University Bochum, Bochum, Germany; Department of Neurology, St. Josef Hospital, Ruhr-University Bochum, Bochum, Germany; Department of Neurology, St. Josef Hospital, Ruhr-University Bochum, Bochum, Germany; Department of Neurology, St. Josef Hospital, Ruhr-University Bochum, Bochum, Germany; Department of Neurology, St. Josef Hospital, Ruhr-University Bochum, Bochum, Germany; Department of Neurology, St. Josef Hospital, Ruhr-University Bochum, Bochum, Germany; Department of Neurology, St. Josef Hospital, Ruhr-University Bochum, Bochum, Germany; Department of Neurology, St. Josef Hospital, Ruhr-University Bochum, Bochum, Germany; Department of Neurology, St. Josef Hospital, Ruhr-University Bochum, Bochum, Germany; Department of Neurology, St. Josef Hospital, Ruhr-University Bochum, Bochum, Germany; Department of Neurology, St. Josef Hospital, Ruhr-University Bochum, Bochum, Germany; Department of Neurology, Augusta Clinic, Hattingen, Germany; Department of Neurology, St. Josef Hospital, Ruhr-University Bochum, Bochum, Germany; Department of Neurology, St. Josef Hospital, Ruhr-University Bochum, Bochum, Germany; Department of Neurology, St. Josef Hospital, Ruhr-University Bochum, Bochum, Germany; Neurodegeneration Research, Centre for Protein Diagnostics (ProDi), Ruhr University, Bochum, Germany; Department of Neurology, St. Josef Hospital, Ruhr-University Bochum, Bochum, Germany

**Keywords:** peripheral neuropathy, high resolution nerve ultrasound, nerve conduction study, head-up tilt test, Parkinson’s disease

## Abstract

The aim of this prospective study was to investigate autonomic function in Parkinson’s disease with a multidimensional approach including clinical evaluation tools, head-up tilt test and morphological studies of the vagus nerve. Head-up tilt test parameters including high frequency power of the heart frequency interval, the ratio of low frequency power of the distance between two consecutive R waves in electrocardiogram (RR interval) to the high frequency and low frequency power of systolic blood pressure were used to evaluate parasympathetic, cardiac sympathetic and vasomotor sympathetic functions, respectively, in 80 patients with Parkinson's disease. We examined the cross-sectional area of the vagus nerves bilaterally using nerve ultrasound and compared mean values with a control group of healthy subjects (*n* = 40) as well as patients with chronic inflammatory demyelinating polyneuropathy (*n* = 76). The cross-sectional area of right/left vagus nerve of Parkinson's patients was significantly lower compared to the right/left vagus nerve of the control group and of chronic demyelinating polyneuropathy patients. Furthermore, the cross-sectional area of the right vagus nerve was significantly larger from the one of the left vagus nerve for all groups. Based on tilt test, 43 patients (disease duration 7 ± 5, age at evaluation 71 ± 9, Hoehn and Yahr score 2.8 ± 8) were diagnosed with autonomic dysfunction (orthostatic hypertension *n* = 11, chronotropic incompetence *n* = 31, postural orthostatic tachycardia syndrome *n* = 1). Patients with orthostatic hypotension showed significantly higher Unified Parkinson’s Disease Rating Scale-III values than those with chronotropic incompetence. The cross-sectional area of the vagus nerve correlated inversely with heart rate in rest and supine position and positively with tilt test parameters representing parasympathetic modulation through vagal activity [high frequency power of the distance between two consecutive R waves in electrocardiogram (RR interval)] at rest. We demonstrate for the first time that morphological characteristics of the vagus nerve correlate with parameters of parasympathetic function from the spectral analysis of cardiovascular parameters in tilt test for Parkinson's patients. This correlation reveals the impact of the atrophy of vagal atrophy for autonomic function in Parkinson's disease. Nerve ultrasound of the vagus nerve could potentially be used as an adjunct to tilt table examination to diagnose autonomic dysfunction.

## Teaser

Huckemann *et al*. report that the mean and right cross-sectional area of the vagus nerve in patients with Parkinson's syndrome was reduced compared to the control group and patients with inflammatory demyelinating neuropathy as a sign of degeneration. Furthermore, it correlated with markers of parasympathetic dysfunction in head-up tilt test.

## Graphical Abstract

**Graphical abstract:**
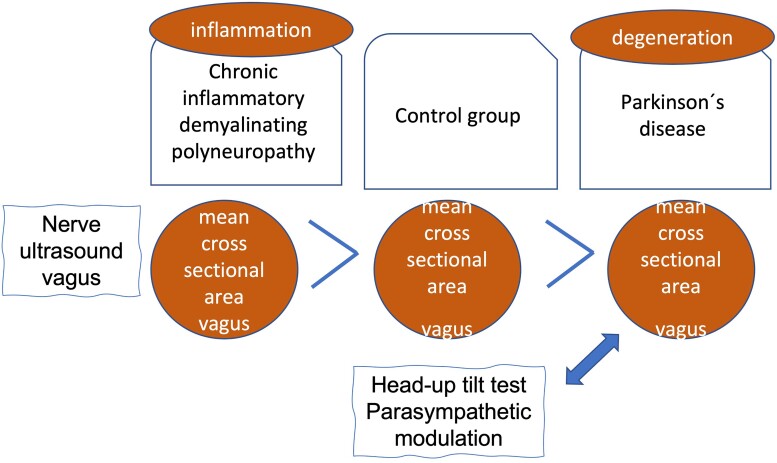

## Introduction

Neurodegenerative disorders with Parkinsonism are characterized by the presence of both motor- and non-motor symptoms including autonomic dysfunction. Autonomic symptoms comprise cardiovascular dysregulation, gastrointestinal symptoms, urinary dysfunction and heat intolerance. They may occur at any stage and impact life quality significantly, especially at more advanced stages.^1-5^

According to a meta-analysis by Velseboer *et al.*, the point prevalence of orthostatic hypotension (OH) in Parkinson's disease (PD) amounts to 30%, other reports revealed even higher ratings due to heterogenous testing conditions and diagnostic criteria.^6^

Previous studies on cardiovascular dysregulation described OH, reduced heart rate variability, supine hypertension and a loss of baroreceptor sensitivity in patients with PD.^7,8^ In cases of PD, OH is attributed to an insufficient norepinephrine release, caused by a denervation of postganglionic sympathetic nerve ends in the myocardium.^8,9^

On the other hand, the reduction or loss of sympathetic skin response (SSR), which has also been demonstrated in PD, confirms preganglionic sympathetic dysfunction. As SSR changes correlated with the severity of motor symptoms, they can also be used as a complementary tool for evaluating autonomic involvement in PD.^10^

Head-up tilt testing (HUTT) provides a non-invasive and standardized diagnostic tool, based on analyses of cardiovascular regulatory mechanisms of heart rate (HR) and blood pressure (BP) changes. It is particularly used for detecting the presence of OH, chronotropic incompetence and postural tachycardia syndrome, which can be provoked by a postural challenge. As cardiovascular regulation is influenced by complex modulatory effects of sympathetic and parasympathetic nerval activities, a sole analysis of HR and BP might be insufficient. Thus, spectral analyses of HR variability and RR-interval variability, a method which has already been used in the context of PD, can provide additional and accurate information on parasympathetic, cardiac sympathetic and vasomotor sympathetic functions.^11^

On the other hand, the nerve ultrasound (NUS) is a common method for investigating morphological changes of peripheral nerves. Latest research has revealed that morphological anomalies of the vagus nerve using NUS are present in patients with PD. Previously obtained data reported a bilateral atrophy of the vagus nerve in PD compared to age-matched controls, whereas morphological changes of the nerve calibres could not be found in phrenic nerves and spinal accessory nerves.^12-14^

Only one study investigated the interdependency between blood pressure and morphological changes of the vagus nerve, but its impact was limited by a small proband size and methods.^12^ Additionally, in cases of diabetic neuropathy and amyotrophic lateral sclerosis, a vagal atrophy could also be found.^15,16^

On the contrary, in cases of Guillan-Barré Syndrom (GBS),^17-19^ an increase of the CSA of the vagus nerve has been detected already in early stages of disease and patients with chronic inflammatory demyelinating polyneuropathy also seem to have slightly higher CSA of the vagus nerve compared to healthy controls in one study probably as a sign of local inflammation.^17,20^ In this case, autonomic dysfunction is attributed to antibody-mediated autoimmunity against sympathetic neurons and consecutive changes in norepinephrine levels.^21,22^

The relevance of the vagus nerve atrophy in PD is still not completely understood. Based on topographical distribution of α-synuclein-enriched Lewy bodies, which could be found in the vagus nerve and the dorsal nuclei of the vagus (dmX) in the lower brainstem,^23^ it is considered that PD has its origin in the gastrointestinal tract and α-synuclein accesses the CNS via retrograde transport.^24^ Furthermore, an asymmetry in size of the right and the left vagus nerve in PD, but also in controls, is described in several studies. This nerve asymmetry is discussed to be caused by anatomical and functional differences between the right and the left vagus nerve.^12^ Still, evidence for a clinical correlation between the morphological characteristics of the vagus nerve, clinical autonomic scores and tilt test data has yet to be found.

Hence, we used NUS for the first time to provide data on morphological changes of the vagus nerve and correlations with markers of parasympathetic dysfunction from HUTT.

## Methods

### Study protocol

We conducted a prospective clinical, sonographical and HUTT study of a cohort of 80 patients with idiopathic PD. 80 of these patients received a sonographical examination of the vagus nerve and 69 of these patients additionally received a HUTT. Due to disease severity as well as technical issues, data acquisition was incomplete for HUTT in 18 cases.

Our study protocol was approved by the local university ethics committee (Medical Faculty of Ruhr University Bochum, Reg. Nr. 18-6360) and is listed in the German Clinical Trials Register (DRKS-ID DRKS00020752). The patients were recruited from the inpatient and outpatient clinic of St Josef Hospital, Bochum. All patients signed informed consent. The study was conducted in accordance with the ethical standards laid down in the declaration of Helsinki of 1964 and its later amendments.

Included in our study were at least 18-year old patients with a diagnosis of idiopathic PD according to the United Kingdom Parkinson's Society Brain Bank criteria and Movement Disorders Society's Criteria.^25,26^ Subjects, who were affected by other established causes of neuropathy (including diabetes mellitus and history of alcohol abuse) or conditions with potential effects on the autonomic nerve system, were excluded from the study.

We retrospectively used an age-matched control group (*n* = 40, female = 19, male = 21), which were examined sonographically in our clinic for other indications non-related with polyneuropathy. For this control group, we excluded all established causes of polyneuropathy.

Furthermore, we included patients with chronic inflammatory demyelinating polyneuropathy from the prospective INHIBIT (Immune-mediated Neuropathies Biomaterial and Data Register) Registry (*n* = 76, female = 20; male = 56 vote-no. 18-6534-BR, registered DRKS00024494), who received examination of the vagus nerve on both sides using the same protocol described for idiopathic PD patients and healthy controls.

The clinical and paraclinical evaluation for the idiopathic PD patients was performed in the following steps.

First, the patients’ demographical data were collected (first diagnosis, disease duration and Hoehn and Yahr stage).

Second, the patients received a clinical examination including patient interview, neurological examination and documentation of the following scores (performed by EK, PA).

MDS-Unified Parkinson’s Disease Rating Scale (MDS-UPDRS) Part I-IV^27^ and Parkinson’s Disease Questionnaire (PDQ-39).^28^

To further evaluate the autonomic symptoms, we used the Assessment of Autonomic Dysfunction in Parkinson’s Disease (SCOPA-AUT).^29^

Third, we conducted HUTT examination and analysed the results using two different methods. First, we evaluated the presence of defined changes: orthostatic hypotension, postural tachycardia syndrome and chronotropic incompetence. Additional haemodynamic parameters were derived by utilizing spectral analysis of blood pressure (BP) and RR interval. We correlated them with the vagus nerve CSA as described in detail below (performed by SH, KP).

Lastly, the vagus nerve on both sides was investigated with NUS as described below in detail (performed by SH). Nerve ultrasound, HUTT testing and clinical evaluation were performed on the same day. Furthermore, we acquired age-matched data from our neurological laboratory in order to generate normal values of the vagus nerve. Due to typical clinical signs of PD blinding did not seem to be feasible.

### Head-up tilt test

To eliminate influencing factors that could interfere with autonomic testing, HUTT was performed with at least 2 hours of abstinence from food. During the examination, the room temperature was maintained at a comfortable level and care was taken to avoid noise. Subjects with a history of heart disease such as myocardial infarction, heart failure and cardiomyopathy were excluded. After an initial rest period of 10 min in supine position, the patients were passively tilted up to 70° for 10 min and subsequently returned to the horizontal basic position. In order to investigate autonomic function, all subjects underwent a continuous non-invasive arterial blood pressure (BP) and heart rate (HR) monitoring, using a Task Force^®^ Monitor system 3040i (CN System, Graz, Austria). Medications potentially affecting blood pressure or heart rate were not discontinued before HUTT due the severity of disease symptoms for the majority of the patients.

In a first step, all HUTT data were analysed concerning the presence of abnormal haemodynamic responses, including OH, postural orthostatic tachycardia syndrome (POTS) and chronotropic incompetence (CI). OH was defined as a reduction in systolic BP ≥20 mmHg or of the diastolic BP ≥10 mmHg from baseline value whereas CI was considered when there was an absence of HR increase more than 10 bpm or an increase less than 10% of the baseline value within 10 min after tilting. POTS was diagnosed in the case of a sustained increase of HR of at least >30 bpm or a HR to >120 bpm while standing.^7,9^

Subsequent analysis of the data yields variation of BP and HR (RR interval in electrocardiogram), continuous beat-to-beat and oscillatory blood pressure (SBP = systolic BP and DBP = diastolic blood pressure). Moreover, utilization of autoregressive spectral analysis provides the power of lower frequencies (LF, from 0.04 to 0.15 Hz) and higher frequencies (HF, 0.15 to 0.4 Hz) of SBP and DBP as well as of the RR-interval variability.^30-32^ As RR-HF is considered as a marker of the vagal cardiac activity in previous studies and RR-LF is mainly modulated by sympathetic activity of the sinus node, the RR-LF to RR-HF ratio (RR-LF/HF) can be related to the cardiac sympathetic activity.^31-33^ SBP-LF, on the other hand, is used as an index of sympathetic vasomotor control.^34^

### Nerve ultrasound examination

Nerve ultrasound was performed under the supervision of KP with at least 10 years of neuromuscular ultrasound experience (performed by SH, KP). All ultrasound studies were performed with an Affinity^®^70G ultrasound system (Philips, Hamburg, Germany), using an 18-MHz linear array transducer. All patients were investigated in supine position. No additional force was applied other than the weight of the transducer to avert artificial compression of the nerve. Measurements of the cross-sectional area (CSA) were performed at the inner border of the thin hyperechoic epineural rim by continuous tracing as described before.^35^

In order to avoid anisotropy, the transducer was kept perpendicular to the vagus nerve, which was measured bilaterally at the level of the bulbus caroticus, between the common carotid artery and the internal jugular vein (Fig. 2).

### Statistical analysis

Statistical analysis was conducted using Prism 8, GraphPad Software, La Jolla, USA. All values are shown as mean with standard deviations (SD) unless stated otherwise. *P* < 0.005 was regarded as statistically significant. Anderson–Darling and D’ Agostino and Pearson normality test σ were applied to test the distribution of the results. Pearson correlation coefficient r was reported for all correlation analyses. To calculate statistical significance between two groups, non-parametrical Mann–Whitney U test was used.

### Data availability

All data generated for this publication are available through the corresponding authors.

## Results

### Demographical data and clinical scores of autonomic dysfunctions

87 patients with PD aged between 45 and 87 years old (mean age 69 ± 11 years old, female *n* = 37, male *n* = 50) were included in the study with a disease duration of 1–20 years (mean disease duration 6 ± 6 years) (Table 1). 80 of these patients received a sonographical examination of the vagus nerve. The clinical characteristics of PD and chronic inflammatory demyelinating polyneuropathy (CIDP) patients, as well as from the control group are depicted in Table 1.

**Table 1:** Demographical and epidemiological characteristics of the patients with Parkinson’s disease (PD) and chronic inflammatory demyelinating polyneuropathy (CIDP) and mean values of the cross-sectional area (CSA) of the vagus nerve. All values are provided as mean ± standard deviation (SD). INCAT/ODSS, inflammatory neuropathy cause and treatment/overall disability sum score, MDS-UPDRS III, MDS-Unified Parkinson’s Disease Rating Scale, SCOPA-AUT, Scales for Outcomes in Parkinson’s Disease- Autonomic Dysfunction, PDQ39, Parkinson’s Disease Questionnaire, CSA = cross-sectional area

	PD	CIDP	Controls	*P*-values
Total (female, male)	80 (37, 50)	76 (20,56)	40 (19,21)	
Age at evaluation (years)	69 ± 11	59 ± 13	65 ± 13	0.1467 (PD versus Controls); 0.0536 (CIDP versus controls); 0.0001 (PD versus CIDP)
Disease duration (years)	6 ± 6	4 ± 4		
Age at diagnosis (years)	63 ± 10	56 ± 13		
Hoehn and Yahr score//INCAT/ODSS	2.7 ± 0.9	3.2 ± 1.7		
MDS-UPDRS III score	30 ± 16			
Scopa-Aut score	14 ± 9			
PDQ39 score	40 ± 30			
Right vagus nerve (CSA mm²)	2.2 ± 0.6	2.9 ± 1.2	2.5 ± 0.6	0.0011 (PD versus Controls); ≤ 0.0001 (PD versus CIDP)
Left vagus nerve (CSA mm²)	2.0 ± 0.6	2.3 ± 1.0	2.1 ± 0.5	0.005 (PD versus Controls)
Vagus nerve mean (CSA mm²)	2.1 ± 0.5	2.6 ± 1.0	2.3 ± 0.5	0.0002 (PD versus CIDP); 0.0005 (PD versus control)

One patient was excluded from the study as a monoclonal gammopathy of undefined significance (IgM kappa) with an underlying demyelinating neuropathy was diagnosed.

### Sonomorphological data of the vagus nerve

NUS revealed a significant reduction of the mean CSA of the vagus nerve in PD patients (*n* = 80) compared to the age-matched controls (*n* = 40) (*P* = 0.0005).

**Figure 1:**
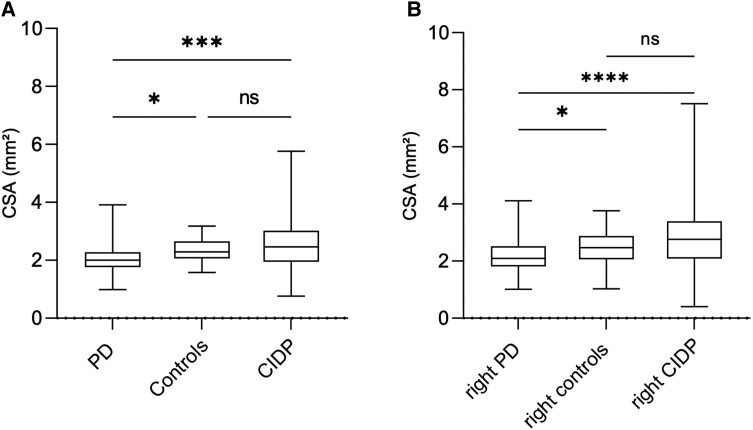
**Mean cross-sectional area values of (A) the vagus nerve for PD, CIDP and control patients and (B) the right vagus nerve of IPS and CIDP patients as well as from the controls.** (Normality tests: Anderson–Darling D’Agostino and Pearson; Kruskal–Wallis test: PD versus controls, *P* = 0.0132; Controls versus CIDP, *P* > 0.9999; PD versus CIDP, *P* = 0.0001). Abbreviations: ns = not significant, *P* > 0.05, *P ≤ 0.05, **P ≤ 0.01, ***P ≤ 0.001, ****P ≤ 0.0001. (Normality tests: Anderson–Darling, D’Agostino and Pearson, Kruskal–Wallis test: right PD versus right controls, *P* = 0.0212; right PD versus right CIDP, *P* < 0.0001; right controls versus right CIDP, *P* = 0.6787; Abbreviations: ns = not significant, *P* > 0.05, *P ≤ 0.05, **P ≤ 0.01, ***P ≤ 0.001, ****P ≤ 0.0001).

Compared to the CIDP group and although the CIDP patients (*n* = 76) were younger (Table 1), we found a significant reduction of the vagus nerve (*P* = 0.0002) for PD patients (Fig. 1A). There was no correlation of the CSA of the vagus nerve with age in any of the groups.

In patients with PD as well as in the other groups, the CSA of the right vagus nerve was significantly larger than the left one (Fig. 1B) (*P* [CIDP] = 0.0004; *P* [controls] = 0.0157).

### Differences concerning CSA of the vagus nerve in PD, CIDP and controls

Using the Mann-Whitney U test, Patients with PD had a lower mean CSA of the vagus nerve compared to the control group and the CIDP group (2.1 ± 0.5 mm² versus 2.3 ± 0.5 mm², *P* = 0.0005; 2.1 ± 0.5 mm² versus 2.6 ± 1.0 mm², *P* = 0.0002). Patients with CIDP had higher CSA from both other groups (2.6 ± 1.0 mm² versus 2.1 ± 0.5 mm², *P* = 0.0002 versus 2.3 ± 0.5 mm², *P* = 0.2436). Furthermore, the CSA of the right vagus nerve was higher in comparison to the left vagus nerve in PD as well as in CIDP patients and controls (PD, 2.2 ± 0.6 mm² versus 2.0 ± 0.6 mm², *P* = 0.0064; CIDP, 2.9 ± 1.2 mm² versus 2.3 ± 1.0 mm², *P* = 0.0004; controls, 2.5 ± 0.6 mm² versus 2.1 ± 0.5 mm², *P* = 0.0157) (Table 1, Supplemental Fig. 1).

Using Kruskal–Wallis test, significant differences between the mean CSA of the vagus nerve of the PD group compared to the control group and the CIDP group could be found (*P* = 0.0132, *P* = 0.0001) but not between controls and CIDP patients (*P* > 0.9999). Furthermore, the CSA of right vagus nerve and not of the left vagus nerve was higher in comparison to the right vagus nerve in both other groups (PD versus controls, *P* = 0.0212; PD versus CIDP, *P* < 0.0001).

**Figure 2:**
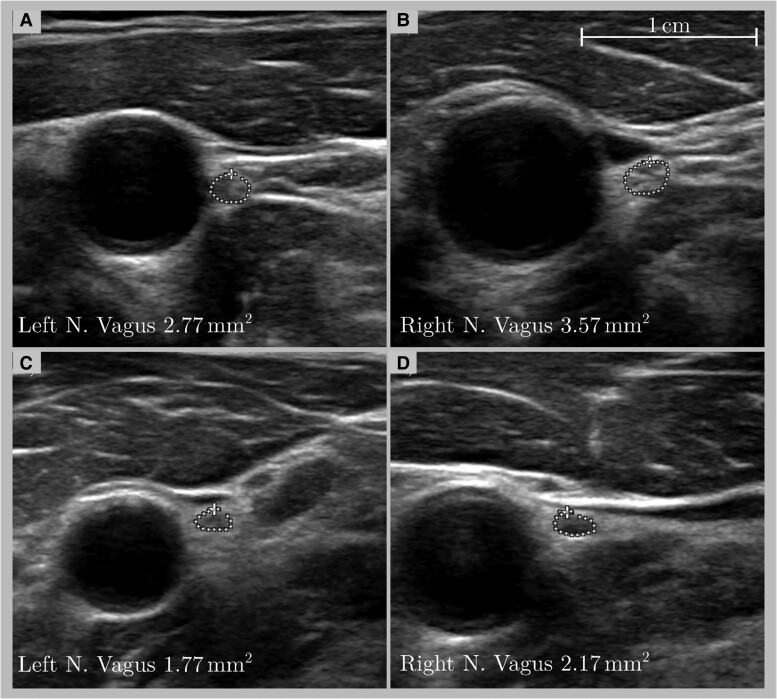
Representative HRUS findings of the vagus nerve in a healthy control subject (A, B) and a patient with PD (C, D) visualized between the common carotid artery and the jugular vein (scale bar 1 cm).

Representative ultrasound pictures of the vagus nerves for all groups are found in Fig. 2.

### HUTT data—patients groups

Based on the HUTT data, 62% of the PD patients presented with autonomic dysfunction (*n* = 43), including CI (*n* = 31), OH (*n* = 11) and POTS (*n* = 1) (Table 2). In our study, significant differences concerning the UPDRS III value between subjects with OH and CI were revealed (43 ± 15 versus 31 ± 14, *P* = 0.025). In addition, patients with CI showed a shorter duration of disease (6 ± 5) compared to those with OH (9 ± 6). The sonographical and epidemiological data are shown in Table 2.

**Table 2:** Epidemiological data of patients with idiopathic Parkinson syndrome based on the table tilt test. All values are provided as mean ± SD. HUTT, head-up table tilt, OH, orthostatic hypotension, CI, chronotropic incompetence, POTS, postural tachycardia syndrome, MDS-UPDRS III, MDS-Unified Parkinson's Disease Rating Scale, SCOPA-AUT, Scales for Outcomes in Parkinson's Disease-Autonomic Dysfunction, PDQ39, Parkinson's Disease Questionnaire, CSA, cross-sectional area. Statistical significance was tested with Mann-Whitney test. MDS-UPDRS-III values in OH were significantly higher than in the CI group. (P = * 0.025)

	Normal HUTT	Pathological HUTT	OH	CI	POTS
**Total (female, male)**	26 (14,12)	43 (17, 26)	11 (2, 9)	31 (15, 16)	1 (0,1)
**Age at evaluation (years)**	66 ± 12	71 ± 9	74 ± 8	70 ± 9	64.2
**Disease duration (years)**	5 ± 6	7 ± 5	9 ± 6	6 ± 5	8
**Age at diagnosis (years)**	60 ± 10	64 ± 8	64 ± 7	64 ± 8	56
**Hoehn and Yahr score**	2.4 ± 0.8	2.8 ± 0.8	2.9 ± 0.7	2.7 ± 0.8	3
**MDS-UPDRS III**	22 ± 11	35 ± 15	43 ± 15	31 ± 14	36
**Scopa-Aut score**	9 ± 6	15 ± 8	17 ± 7	14 ± 9	22
**PDQ39 score**	24 ± 21	49 ± 29	53 ± 24	50 ± 40	52
**Right vagus nerve in HRUS (CSA mm²)**	2.2 ± 0.6	2.2 ± 0.6	2.4 ± 0.6	2.2 ± 0.6	1.8
**Left vagus nerve in HRUS (CSA mm²)**	2.0 ± 0.7	2.1 ± 0.7	2.4 ± 0.8	1.9 ± 0.5	2.1
**Mean vagus nerve in HRUS (CSA mm²)**	2.1 ± 0.6	2.1 ± 0.6	2.4 ± 0.6	2.1 ± 0.5	2

### Correlation of the HUTT with clinical parameters

We next performed correlation of the HUTT parameters with clinical and autonomic scores included in the study and reported above. No significant correlations were found between clinical parameters and HUTT (data not shown).

### Correlation of the HUTT with CSA of the vagus nerve

Next, we performed correlation analyses of the CSA of the vagus nerve with the following parameters (Table 3): HR in supine position, HR in rest, RR-HF is considered as a marker of the vagal cardiac activity, RR-LF is mainly modulated by sympathetic activity of the sinus node, the RR-LF to RR-HF ratio (RR-LF/HF) can be related to the cardiac sympathetic activity.

**Table 3:** Correlations between cross-sectional area of the vagus nerve and tilt test parameters in rest, after tilting in upright position and in supine position, representing sympathetic and parasympathetic activity. The table contains mean values as well as standard deviation. HR, heart rate, LFnu-RRI, low frequencies power of the RR Interval (normalized units); LF-RRI, low frequencies power of the RR Interval; HFnu-RRI, high frequencies power of the RR Interval (normalized units); HF-RRI, high frequency power of RR Interval; LF/HF-RRI, ratio of low frequency power of RR interval to RR-HF; LF/HF, ratio of the LF to HF; LF/HF-dBP, ratio of low frequency to high frequency power of diastolic blood pressure.

		Vagus right		Vagus left		Vagus mean	
		r	*P*	r	*P*	r	*P*
**Vagus nerve CSA**	Right vagus			0.508	0.0000	0.867	0.00
	Left vagus	0.508	0.0000			0.850	0.0000
	Vagus mean	0.867	0.0000	0.850	0.0000		
**HR**	HR rest	−0.352	0.0040	−0.027	0.8318	−0.234	0.06095
	HR upright	−0.249	0.0452	−0.109	0.38678	−0.209	0.09406
	HR supine	−0.399	0.0013	−0.122	0.34538	−0.304	0.01643
	Modulated by sympathetic activity of the sinus node						
**LFnu**-**RRI**	LFnu-RRI rest	−0.292	0.0213	−0.168	0.1932	−0.268	0.035
	LFnu-RRI upright	−0.317	0.0122	−0.109	0.3984	−0.248	0.052
	LFnu-RRI supine	−0.153	0.2469	−0.064	0.6295	−0.132	0.319
**LF**-**RRI**	LF-RRI rest	0.144	0.2654	0.281	0.0270	0.254	0.0467
	LF-RRI upright	0.049	0.7041	0.237	0.0636	0.168	0.1912
	LF-RRI supine	0.031	0.8155	0.135	0.3069	0.118	0.3745
	Parasympathetic modulation marker of vagal cardiac activity						
**HFnu**-**RRI**	HFnu-RRI rest	0.279	0.0279	0.183	0.1540	0.269	0.0346
	HFnu-RRI upright	0.378	0.0025	0.204	0.1126	0.330	0.0089
	HFnu-RRI supine	0.221	0.0932	0.161	0.2230	0.219	0.0959
**HF-RRI**	HF-RRI rest	0.312	0.0136	0.304	0.0164	0.356	0.0045
	HF-RRI upright	0.224	0.0797	0.245	0.0546	0.271	0.0331
	HF-RRI supine	0.157	0.2350	0.193	0.1440	0.216	0.1008
	Referred as cardiac sympathetic activity						
**LF/HF**-**RRI**	LF/HF-RRI rest	−0.281	0.0271	−0.177	0.1686	−0.265	0.0372
	LF/HF-RRI upright	−0.326	0.0097	−0.148	0.2512	−0.265	0.0374
	LF/HF-RRI supine	−0.237	0.0707	−0.161	0.2220	−0.232	0.0769
**LF/HF**	LF/HF rest	−0.239	0.0641	−0.128	0.3250	−0.201	0.1212
	LF/HF upright	−0.404	0.0013	−0.187	0.1496	−0.335	0.0084
	LF/HF supine	−0.259	0.0500	−0.049	0.7173	−0.181	0.1737
**LF/HF-dBP**	LF/HF-dBP rest	−0.205	0.1068	−0.198	0.1195	−0.219	0.0850
	LF/HF-dBP upright	−0.366	0.0032	−0.288	0.0219	−0.375	0.0024
	LF/HF-dBP supine	−0369	0.0038	−0.294	0.0224	−0.383	0.0025

**Figure 3:**
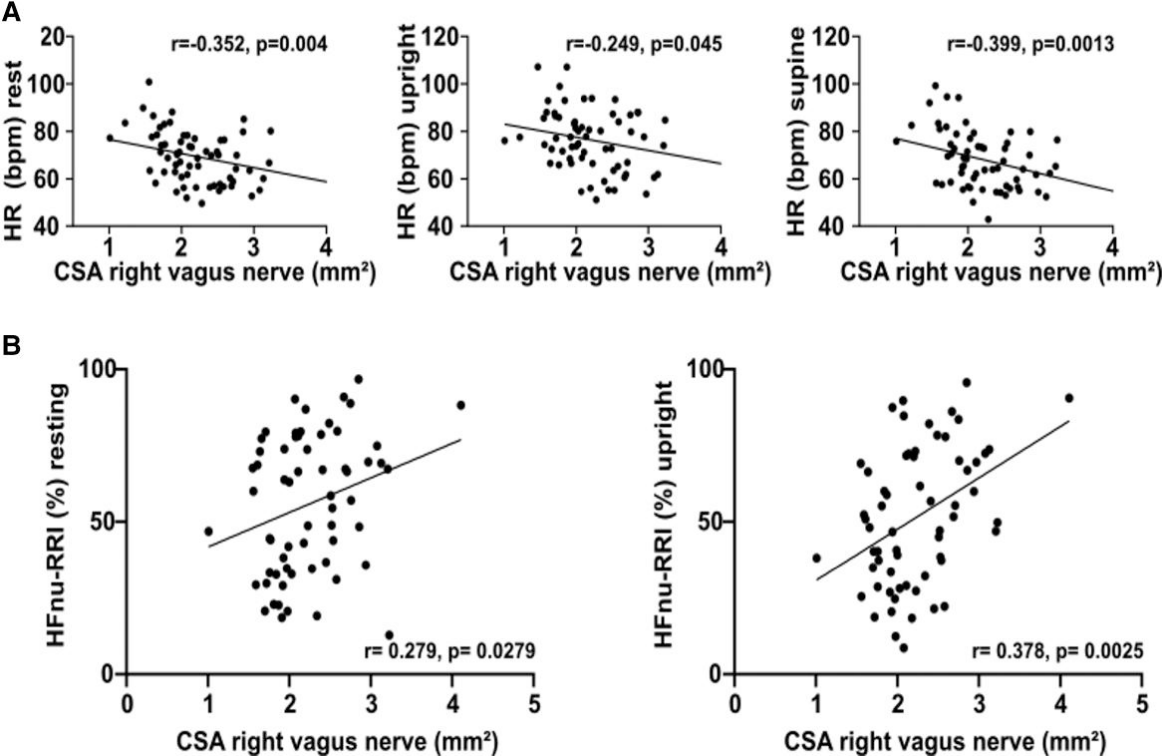
**Schematic presentation of the correlation of the HUTT with the CSA of the vagus nerve.** (**A**) Correlation of CSA of the right vagal nerve in mm² and the HR before tilting, after tilting in upright position and in supine position. (**B**) Correlation of the CSA of vagal nerve in mm² and markers of parasympathetic activity (HFnu-RRI) in rest (r = 0.279, *P* = 0.0279) and upright position (r = 0.378, *P* = 0.0025). Abbreviations: HR = heart rate, bpm = beats per minute, CSA = cross-sectional area and HFnu-RRI = high frequencies power of the RR Interval.

The CSA of the right vagus nerve correlated inversely with HR in rest, upright and supine position. A positive correlation was found between the right vagus nerve with parameters representing parasympathetic modulation through vagal activity (HF-RRI) at rest and concerning the HFnu-RRI also in rest and after tilting. The left vagus nerve correlated significantly with the HF-RRI and the LF-RRI in resting position (Table 3, Fig. 3). No further significant correlation was found between CSA of the vagus nerve and clinical autonomic parameters (data not shown).

## Discussion

In this study, we have detected significant morphological differences of the CSA of the vagus nerve between CIDP, PD and a healthy control group. Evidence from previous clinical trials of a reduction in mean and right vagus nerve CSA was confirmed in our study of a larger cohort of patients with IPS.^12,14^ A possible underlying pathophysiological process behind this vagal atrophy is the α-synuclein deposition with consecutive neurodegenerative changes postulated by Braak *et al.*^23^ In addition, a significant asymmetry between the right and the left vagus nerve was found (right vagus nerve was larger than the left vagus nerve), consistent with other study results.^12^Anatomical and functional differences in the innervation area between the right and left vagus nerves are discussed as the cause for this morphological abnormality, which is also detectable in the control and CIDP groups. Compared to the control group, the CIDP patients did not show a significant enlargement of the mean CSA of the vagus nerve (CIDP 2.6 mm² versus 2.3 mm² in controls; *P* = 0,2346). Nevertheless, the mean CSA and CSA of the right vagus nerve were larger in CIDP patients compared to PD (Table 1) although their age was lower.

A significant enlargement of the vagus nerve CSA in early disease stages of GBS was found compared with CIDP; however, a CSA vagus increase in CIDP compared to control has not been reported.^17,20^ In the context of CIDP, an autonomic dysfunction has been described only in rare cases; therefore, a CSA increase of the vagus nerve compared to controls was not expected.^36^

Although NUS has some technical and investigator-dependent limitations, significant differences could be demonstrated between all studied groups. Further longitudinal studies are needed to investigate possible dynamics of vagal nerve cross-sectional area in IPS during the progress of the disease and to evaluate the clinical significance of sonography as a complementary diagnostic method in follow-up.

The high prevalence of pathologic tilt table findings in the cohort of IPS patients (62%) emphasize the clinical relevance of autonomic dysfunction. In our study, significant differences concerning the UPDRS III values between subjects with OH and CI emerged (43 ± 15 versus 31 ± 14, *P* = 0.025). A possible explanation for these differences is that CI may represent a precursor of OH in earlier stages of disease. Further longitudinal studies and a larger number of subjects are needed to evaluate this hypothesis. Nevertheless, the overall shorter duration of disease in patients with CI (6 ± 5) compared to those with OH (9 ± 6) argues in favour of this hypothesis.

Surely, one of the shortcomings of the study is that the medication potentially influencing heart rate and blood pressure were not discontinued before HUTT; therefore, the high prevalence of autonomic dysfunction in our PD cohort argues for an even higher prevalence without the stabilizing concomitant medication.

For the first time, we demonstrated that anatomical characteristics of the vagus nerve correlate with parameters of parasympathetic function from the spectral analysis of cardiovascular parameters in HUTT for PD. In this regard, we found that the right and left vagus nerve does not correlate equally with the tilt table parameters collected. Pronounced correlations were more apparent for the right vagus nerve. This result is consistent with the findings of Walter *et al.* whose study, however, is limited by a restricted number of subjects and investigative techniques.^12^ This correlation enables a new interpretation of the anatomical characteristics of the vagus nerve for PD patients and diagnosis of autonomic dysfunction possibly implying that vagus nerve atrophy indicates parasympathetic dysfunction. Therefore, NUS of the vagus nerve could potentially be used as an adjunct to tilt table examination to diagnose autonomic dysfunction, whereas CSA of the vagus nerve can be used as a marker of autonomic disease progression in PD remains to be investigated in longitudinal studies.

Concluding, we present the first detailed study on vagus sonographical characteristics focusing on vagal atrophy for PD. These findings present a novel role for nerve ultrasound of the vagus nerve as a marker of autonomic dysfunction and reveal the need for further longitudinal studies to investigate the development of these changes over the years.

## Competing interests

All authors report no conflicts of interest regarding this manuscript.

## Supplementary Material

fcad006_Supplementary_Data

## Abbreviations

BP =: Blood pressure
Bpm =: beats per minute
CI =: chronotropic incompetence
CIDP =: chronic inflammatory demyelinating polyneuropathy patients
DBP =: diastolic blood pressure
GBS =: Guillan-Barré Syndrom
HF =: High Frequency
HF-RRI =: high frequency power of RR Interval
HFnu-RRI =: high frequencies power of the RR Interval (normalized units)
HR =: Heart rate
HUTT =: Head-up tilt testing
INCAT/ODSS =: inflammatory neuropathy cause and treatment/overall disability sum score
LF =: Low frequency
LF-RRI =: low frequencies power of the RR Interval
LF/HF =: ratio of the LF to HF
LF/HF-dBP =: ratio of low frequency to high frequency power of diastolic blood pressure
LF/HF-RRI =: ratio of low frequency power of RR interval to RR-HF
LFnu-RRI =: low frequencies power of the RR Interval (normalized units)
MDS-UPDRS =: MDS-Unified Parkinson’s Disease Rating Scale
Ns =: not significant
NUS =: Nerve ultrasound
OH =: orthostatic hypotension
PD =: Parkinsons’s disease
PDQ-39 =: Parkinson’s Disease Questionnaire
POTS =: postural orthostatic tachycardia syndrome
RR =: interval Intervall between R waves in electrocardiogram
SBP =: systolic BP
SCOPA-AUT =: Assessment of Autonomic Dysfunction in Parkinson’s Disease
SD =: Standard deviation
SSR =: sympathetic skin response
