# Supplementary material for: Vagal cross-sectional area correlates with parasympathetic dysfunction in Parkinson's disease
Source: Brain Commun. 2023 Jan 18;5(1):fcad006. doi: 10.1093/braincomms/fcad006 (PMC9883711; doi:10.1093/braincomms/fcad006)
Supplement: fcad006_Supplementary_Data [file fcad006_supplementary_data.pdf]

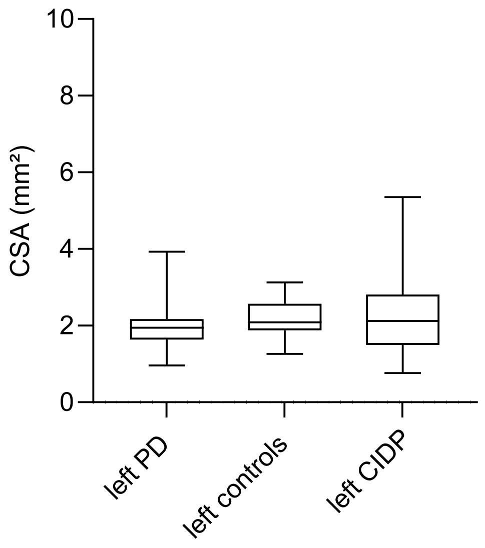

Supplementary figure 1

Mean cross sectional area values of the left vagus nerve of IPS and CIDP patients as well as from the controls

(Normality tests: Anderson-Darling, D’Agostino & Pearson, Kruskal-Wallis test:
Kruskal-Wallis test: left PD vs left controls, P=0.0525 ; left PD vs left CIDP, P= 0.260; left controls vs left CIDP, P=>0.9999)
<u>Abbreviations:</u> ns= not significant, P>0.05, *= P≤ 0.05, **= P≤ 0.01, ***= P≤ 0.001, ****= P≤ 0.0001)
